# Preoperative histogram parameters of dynamic contrast‐enhanced MRI as a potential imaging biomarker for assessing the expression of Ki‐67 in prostate cancer

**DOI:** 10.1002/cam4.3912

**Published:** 2021-06-12

**Authors:** Yongsheng Zhang, Zhiping Li, Chen Gao, Jianliang Shen, Mingtao Chen, Yufeng Liu, Zhijian Cao, Peipei Pang, Feng Cui, Maosheng Xu

**Affiliations:** ^1^ Department of Radiology The Guangxing Hospital Affiliated to Zhejiang Chinese Medical University Hangzhou China; ^2^ Department of Radiology The First Affiliated Hospital of Zhejiang Chinese Medical University Hangzhou China; ^3^ Department of Pathology The First Affiliated Hospital of Zhejiang Chinese Medical University Hangzhou China; ^4^ GE Healthcare Life Sciences Hangzhou China; ^5^ The First Clinical Medical College of Zhejiang Chinese Medical University Hangzhou China

**Keywords:** magnetic resonance imaging, pharmacokinetics, prostatic neoplasms

## Abstract

**Purpose:**

To investigate whether preoperative histogram parameters of dynamic contrast‐enhanced MRI (DCE‐MRI) can assess the expression of Ki‐67 in prostate cancer (PCa).

**Materials and methods:**

A consecutive series of 76 patients with pathology‐proven PCa who underwent routine DCE‐MRI scans were retrospectively recruited. Quantitative parameters including the volume transfer constant (K^trans^), rate contrast (K_ep_), extracellular‐extravascular volume fraction (V_e_), and plasma volume (V_p_) by outlining the three‐dimensional volume of interest (VOI) of all lesions were processed. Then, the histogram analyses of these quantitative parameters were performed. The Spearman rank correlation analysis was used to evaluate the correlation of these parameters and Ki‐67 expression of PCa. Receiver operating characteristic (ROC) curve analysis was adopted to evaluate the efficacy of these quantitative histogram parameters in identifying high Ki‐67 expression from low Ki‐67 expression of PCa.

**Results:**

Eighty‐eight PCa lesions were enrolled in this study, including 31 lesions with high Ki‐67 expression and 57 lesions with low Ki‐67 expression. The median, mean, 75th percentile, and 90th percentile derived from K^trans^ and K_ep_ had a moderately positive correlation with Ki‐67 expression (*r* = 0.361–0.450, *p *< 0.05), in which both the median and mean of K^trans^ had the highest positive correlation (*r* = 0.450, *p *< 0.05). The diagnostic efficacy of the K^trans^ median, mean, 75th percentile, and 90th percentile, along with the K_ep_‐based median and mean was assessed by the ROC curve. The area under the curve (AUC) of the mean for K^trans^ was the highest (0.826). When the cut‐off of the mean for K^trans^ was ≥0.47/min, its Youden index, sensitivity, and specificity were 0.625, 0.871, and 0.754, respectively. The AUC of the median of K_ep_ was the lowest (0.772).

**Conclusion:**

The histogram of DCE‐MRI quantitative parameters is correlated with Ki‐67 expression, which has the potential to noninvasively assess the expression of Ki‐67 with patients of PCa.

## Graphical abstract

In this work, we found that the median, mean, 75th percentile, and 90th percentile derived from Ktrans and Kep had a moderately positive correlation with Ki‐67 expression. We also chose the histogram quantitative parameters with an r value ≥0.4 to discriminate high Ki‐67 expression from low Ki‐67 expression in PCa. And six histogram parameters derived from Ktrans and Kep were selected, in which the AUC of mean for Ktrans was the highest (0.826).

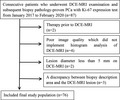

## 1 INTRODUCTION

Prostate cancer (PCa) is the second most frequently diagnosed cancer in men worldwide.^1^ Currently, treatment methods for PCa include prostatectomy, antihormonal therapy, radiation, cryotherapy, active surveillance, and so on. Early detection, localization, and tumor staging have substantially significant for PCa treatment choice. A reliable, prognostic biomarker may be an aid to adopt individual and precise treatment and avoid unnecessary treatments.^2^

The Ki‐67 nucleoprotein is a widely used biomarker to measure tumor biological behavior.^3^ The expression level of Ki‐67 in PCa is significantly associated with tumor grading and stage. A meta‐analysis demonstrated that the high Ki‐67 expression was a factor of poor prognosis for disease‐specific survival, biochemical failure‐free survival, disease‐free survival, rate of distant metastases, and overall survival after curative‐intent treatments in localized PCa patients.^4^ As such, the Ki‐67 expression may be used as an independent biomarker for predicting the prognosis of PCa.^3^, ^4^, ^5^, ^6^ However, the Ki‐67 expression can only be determined by immunohistochemistry or reverse transcription‐polymerase chain reaction after transrectal ultrasound (TRUS)‐guided prostate biopsy or prostatectomy. These techniques are invasive, expensive, and time‐consuming. Besides, biopsy samples cannot comprehensively reflect intratumoral heterogeneity expression. Therefore, a noninvasive, accurate measure of Ki‐67 expression would be of great value.

Multiparametric MRI (mp‐MRI) containing T1‐ and T2‐weighted imaging (T1WI and T2WI), diffusion‐weighted imaging (DWI), and dynamic contrast‐enhanced (DCE) has been regarded as the most advanced imaging modality for the detection of PCa.^7^, ^8^ Compared to DWI and T2WI, DCE‐MRI is less commonly used but plays an indispensable role in PCa according to the Prostate Imaging Reporting and Data System Version 2 and 2.1 (PI‐RADS V2 and V2.1).^7^, ^9^ DCE‐MRI has been demonstrated more accurately to analyze PCa lesions by measuring vascular permeability and perfusion.^10^, ^11^, ^12^ DCE‐MRI plays an essential role in the assessment of local recurrence following prior radical treatment or focal therapy. A significant correlation between pharmacokinetic parameters of DCE‐MRI and histopathologic parameters such as microvessel density and Gleason score was found in PCa.^11^, ^12^ Niekerk et al. found that K_ep_ had a significant correlation with microvessel density (*r* = 0.61, *p *= 0.007) and perimeter (*r* = 0.54, *p* = 0.022) by analyzing the ratio between tumor and normal tissue.^12^ However, the traditional parameters of DCE‐MRI do not comprehensively reflect the heterogeneity of tumor blood vessels through their mean and median.^13^ Therefore, the three‐dimensional histogram analysis of DCE‐MRI is a commonly used approach to reflect the distribution of tumor blood vessels.^13^ Histogram parameters derived from DCE‐MRI were associated with microvessel density and Programmed death 1 ligand expression of immune cells in head and neck squamous cell cancer,^14^, ^15^ isocitrate dehydrogenase 1 mutation and vascular endothelial growth factor expression level in glioma,^16^ prognostic factors and molecular subtypes in breast cancer.^17^ Besides, it also can be used to assess chemoradiotherapy treatment response for locally advanced esophageal squamous cell carcinoma ^18^ and predict yttrium 90 radioembolization treatment stratification in hepatocellular carcinoma.^19^

Several previous studies have found that DCE‐MRI could be used to predict the Ki‐67 expression in breast cancer,^20^ soft tissue sarcomas,^21^ head and neck squamous cell cancer,^14^ and hepatocellular carcinoma.^22^ The feasibility and value of histogram analysis derived from DCE‐MRI for predicting the Ki‐67 expression in PCa have not been investigated. Therefore, the purpose of this study was to evaluate the correlation between the histogram analysis of DCE‐MRI and the expression of Ki‐67 in PCa.

## 2 MATERIALS AND METHODS

### 2.1 Patients

**FIGURE 1:**
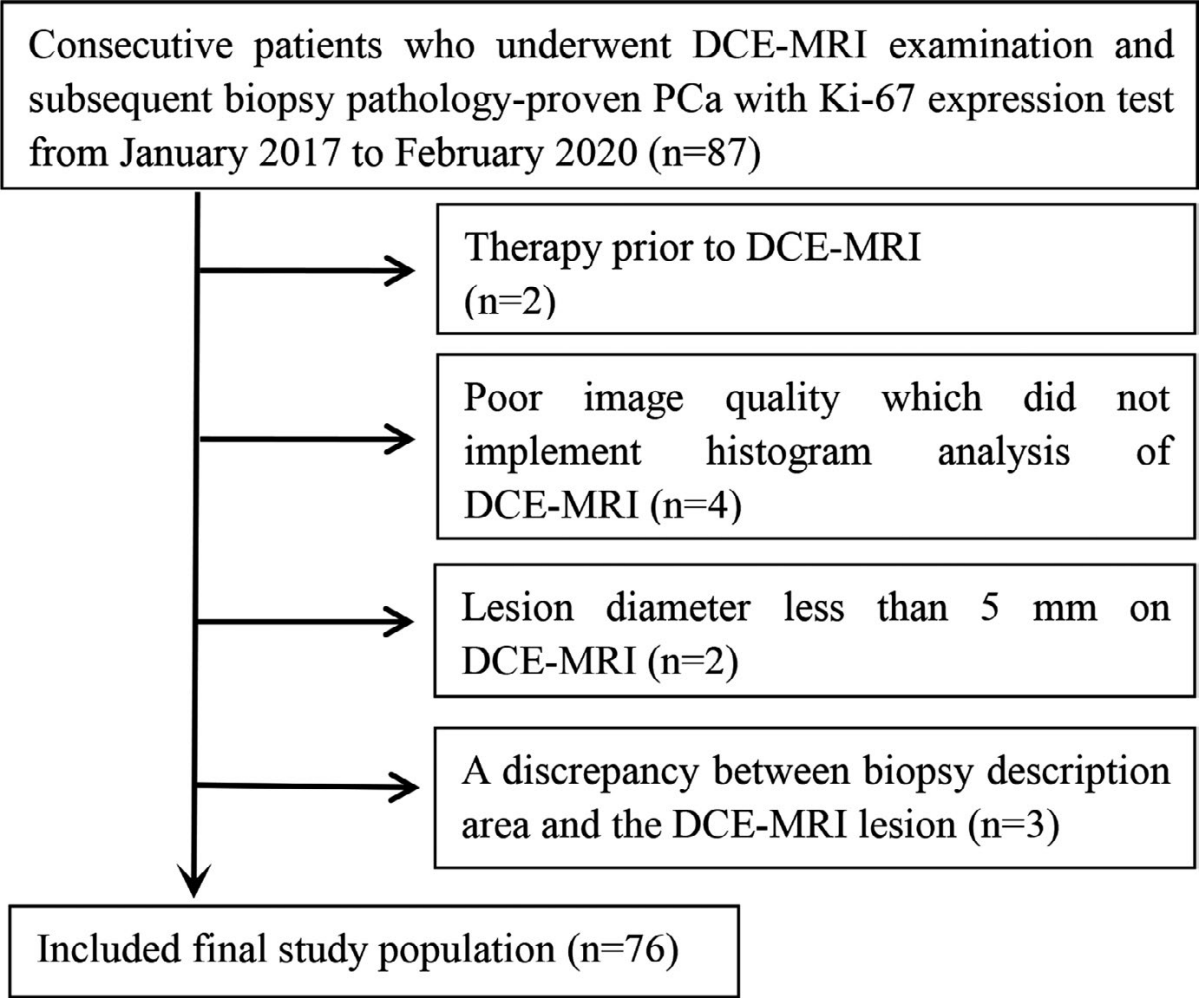
Flowchart displays inclusion and exclusion criteria and the selection process used in this study. Note: DCE‐MRI, dynamic contrast‐enhanced MRI; PCa, prostate cancer

This retrospective analysis was approved by the Institutional Ethical Committee of the First Affiliated Hospital of Zhejiang Chinese Medical University, which waived the requirement for written informed consent. A consecutive series of 87 patients from January 2017 to February 2020 met the following inclusion criteria and were enrolled from the Picture Archiving and Communication Systems of our hospital. All patients met the following criteria; (a) patients with biopsy pathology‐proven PCa; (b) patients who had a prebiopsy 3.0 T MRI with the same MRI scanner; (c) patients who underwent Ki‐67 expression test with complete clinicopathological data. Of the 87 patients, 11 patients were excluded due to the following reasons: (a) prior therapy histories such as antihormonal therapy, radiation, or cryotherapy; (b) poor image quality which did not include histogram analysis of DCE‐MRI; (c) lesion diameter less than 5 mm on DCE‐MRI; (d) a discrepancy between pathological description area and the DCE‐MRI lesion. Ultimately, 76 patients with 88 PCa lesions were recruited in this study (Figure 1).

Baseline clinical features were derived from the Picture Archiving and Communication Systems, including age, Gleason score, and prostate‐specific antigen (PSA) with a cut‐off value of 10 ng/ml. The interval time between DCE‐MRI and PSA examination was less than one month. The lesion's diameter and position were recorded from DCE‐MRI images.

### 2.2 MRI Protocol

All examinations were performed using the same 3.0 T MRI scanner (Discovery 750 W 3.0 T, GE Healthcare, Milwaukee, USA) and a 32 channel pelvic coil. The imaging protocol contained transverse T1WI, axial, sagittal, coronal T2WI, transverse DWI, and DCE. DCE‐MRI was acquired by the Liver Acquisition with Volume Acceleration (LAVA) sequence (TR/TE 2.6/1.1 ms, 3 mm section thickness, 0 mm section gap, 380 mm × 380 mm field of view, 200 × 160 matrix, 12° flip angle, 13.7 s temporal resolution, 4.6 min time of acquisition). Prior to the DCE scan, T1 mapping was acquired with a 5° and 13° flip angle. Subsequently, Gadopentetate dimeglumine injection (contrast medium, Gd‐DTPA, Beilu, China) was administered after two baseline scans through an intravenous bolus injection (0.2 ml/kg body weight) at a rate of 2.5 ml/s using a high‐pressure mechanical injector, followed by a flush of 15 ml of saline solution. The details of all imaging sequence parameters are summarized in Table S1.

### 2.3 Pathological and immunohistochemical analysis

The TRUS‐guided, 10‐core systematic biopsy, and an MRI‐targeted biopsy were performed after the MRI examination for all patients with suspected PCa. A single urologist with 16 years of experience conducted each biopsy and recorded the biopsy position and depth. Ki‐67 was tested by an EnVision Plus kit (Zhongshan, Beijing, China). The antigen‐antibody reaction experiment was conducted according to the kit instructions, in which the mouse anti‐human Ki‐67 antigen immunohistochemistry monoclonal antibody was adopted as the primary antibody. The whole specimens were examined and the expression level of Ki‐67 was assessed under 100‐fold microscopic examination by two genitourinary pathologists to reach a consensus. The corresponding Gleason score was noted. For situations of the discrepancy between the two pathologists, a pathological professor with more than 20 years of experience for PCa evaluated Ki‐67 expression level to break the tie. When there were multiple PCa lesions with different expression levels of Ki‐67, the Ki‐67 expression of each lesion was detected. According to previous studies, the optimal value for high Ki‐67 expression was considered when more than 10% of positive cells were determined.^5^ Therefore, our study cases were divided into two categories: high Ki‐67 expression group (Ki‐67 > 10%) and low Ki‐67 PI expression group (Ki‐67 ≤ 10%).

### 2.4 DCE‐MRI postprocessing and analysis

Two radiologists who had 7 and 11 years of experience in prostate MRI‐delineated PCa lesions by Omni‐Kinetics software (version V2.1.1R, GE Healthcare) to calculate the histogram parameter of DCE‐MRI. The Extended Tofts Linear model was selected as the pharmacokinetic model. The arterial input function (AIF) was performed and the population AIF was derived manually from the femoral artery. The volume of interest (VOI) was delineated along the contour of the tumor. Given the importance of heterogeneity analysis, VOI was designed to contain regions of calcification, necrosis, bleeding, and cystic tissue. Normal anatomic structures were avoided. For varying pathological Gleason scores (GS), each GS lesion was selected for examination. If all lesions demonstrated the same GS, the VOIs were depicted at each level manually until all lesions were incorporated. The T2WI, DWI and apparent diffusion coefficient (ADC) images were also adopted as a reference to better examine the tumor. The concordance of DCE‐MRI lesion and biopsy lesion was evaluated by another radiologist with 21 years of experience of abdominal MRI.

Finally, the quantitative parameters of DCE‐MRI including the volume transfer constant (K^trans^), rate contrast (K_ep_), extracellular‐extravascular volume fraction (V_e_), and plasma volume (V_p_) were obtained and their relationship was examined with the formula (K^trans^ = V_e_ × K_ep_).^12^ Then, the histogram analyses of these quantitative parameters were performed to acquire the minimum, maximum, median, mean, area, 10th percentile, 25th percentile, 75th percentile, and 90th percentile.

### 2.5 Statistical analysis

Continuous variables demonstrated the mean and standard deviation (SD), using the independent‐sample *t*‐test or Mann–Whitney U‐test when appropriate. Categorical variables demonstrate the frequency by performing Fisher's exact test or Chi‐squared test when appropriate. Kolmogorov–Smirnov test was adopted to assess whether the continuous variable was normally distributed. Spearman rank correlation analysis was used to evaluate the correlation between the histogram parameters of DCE‐MRI for PCa and Ki‐67 expression level because the continuous variables are not normally distributed. Positive and negative R values suggest positive and negative correlations, respectively. An R‐value greater than or equal to 0.7 was selected to demonstrate a strong correlation, whereas an R less than or equal to 0.4 demonstrated weak or no correlation.^23^ The receiver operating characteristic (ROC) was used to evaluate the diagnostic efficacy of various histogram quantitative parameters in identifying high Ki‐67 expression (Ki‐67 > 10%) and low Ki‐67 PI expression (Ki‐67 ≤ 10%) of PCa. The parameter with the optimal Youden index was selected and the corresponding sensitivity and specificity were calculated. The correlation scatterplot was utilized with the “ggpubr” package by R software (v. 3.5.1, Vienna, Austria). SPSS 22.0 (SPSS Inc., Chicago, Illinois, USA) and Medcalc 19.0 (MedCalc Software Ltd, Belgium) were performed to implement the remained statistical analysis. A *p* < 0.05 in two‐tailed analyses was used to define statistical significance.

## 3 RESULTS

**FIGURE 2:**
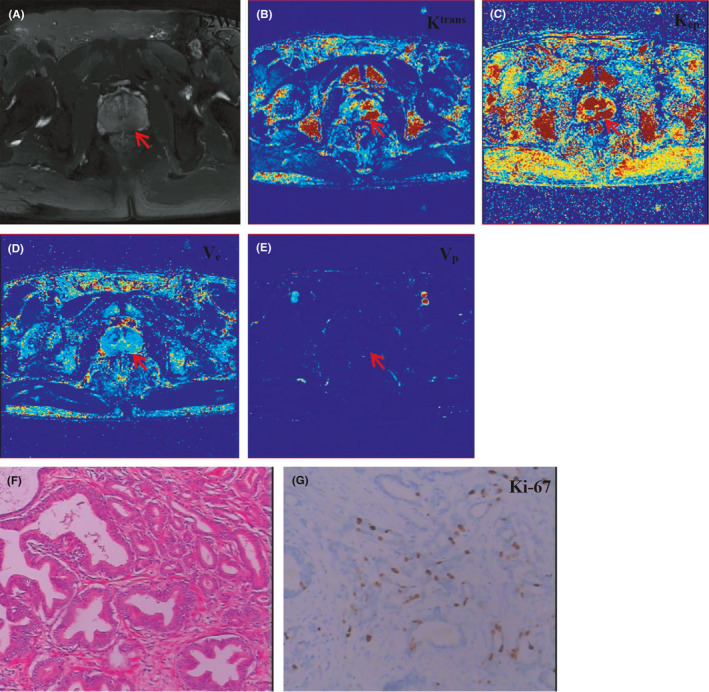
Different imaging modalities demonstrating a single left peripheral prostate cancer from a 62‐year‐old male patient with prostatectomy pathology (Gleason score 3 + 4 and serum PSA 9.89 ng/ml): (A) Transverse T2‐weighted MR image showing a hypointense area with obscure border (red arrow); (B) DCE‐MRI K^trans^ displaying a patchy red region (red arrow); (C) DCE‐MRI K_ep_ displaying a patchy red region (red arrow); (D) DCE‐MRI V_e_ showing a patchy, slightly blue area (red arrow); (E) DCE‐MRI V_p_ showing a dark blue area (red arrow) that is difficult to distinguish; (F) routine pathology (10 × 10); (G) Ki‐67 imaging with 10% positive tumor cells (10 × 10)

Eighty‐eight PCa lesions from 76 patients were enrolled in this study, in which 64 cases had one lesion, and 12 cases had two lesions. The high Ki‐67 expression group had 31 tumor lesions, whereas the low Ki‐67 expression group contained 57 tumor lesions. The characteristics of patients in the high and low Ki‐67 expression groups are shown in Table 1. The age, PSA level, lesion diameter, and lesion's position between the two groups had no statistical difference (*p *> 0.05). The Gleason score was the only parameter to show a statistically significant difference (*p *< 0.05). The example case is displayed in Figure 2.

**TABLE 1:** Characteristics of patients in the high and low Ki‐67 expression groups

Characteristics	High Ki−67 Expression group (*n*=31)	Low Ki−67 Expression group (*n*=57)	*p*
Age (years)	72.230 ± 9.605	72.700 ± 8.736	0.814
PSA (ng/ml)	56.300 ± 133.582	55.700 ± 160.396	0.986
Diameter (mm)	21.410 ± 12.771	18.060 ± 10.064	0.180
Position			0.549
Peripheral zone	21	34	
Transitional zone	8	19	
Peripheral and Transitional zone	2	4	
Gleason score			0.000*
6	NA	20	
7	11	24	
8	8	8	
9	10	5	
10	2	NA	

*n* indicates the number of lesions.

Abbreviations: NA, not available; PSA, prostate‐specific antigen.

* *p* value < 0.05.

**FIGURE 3:**
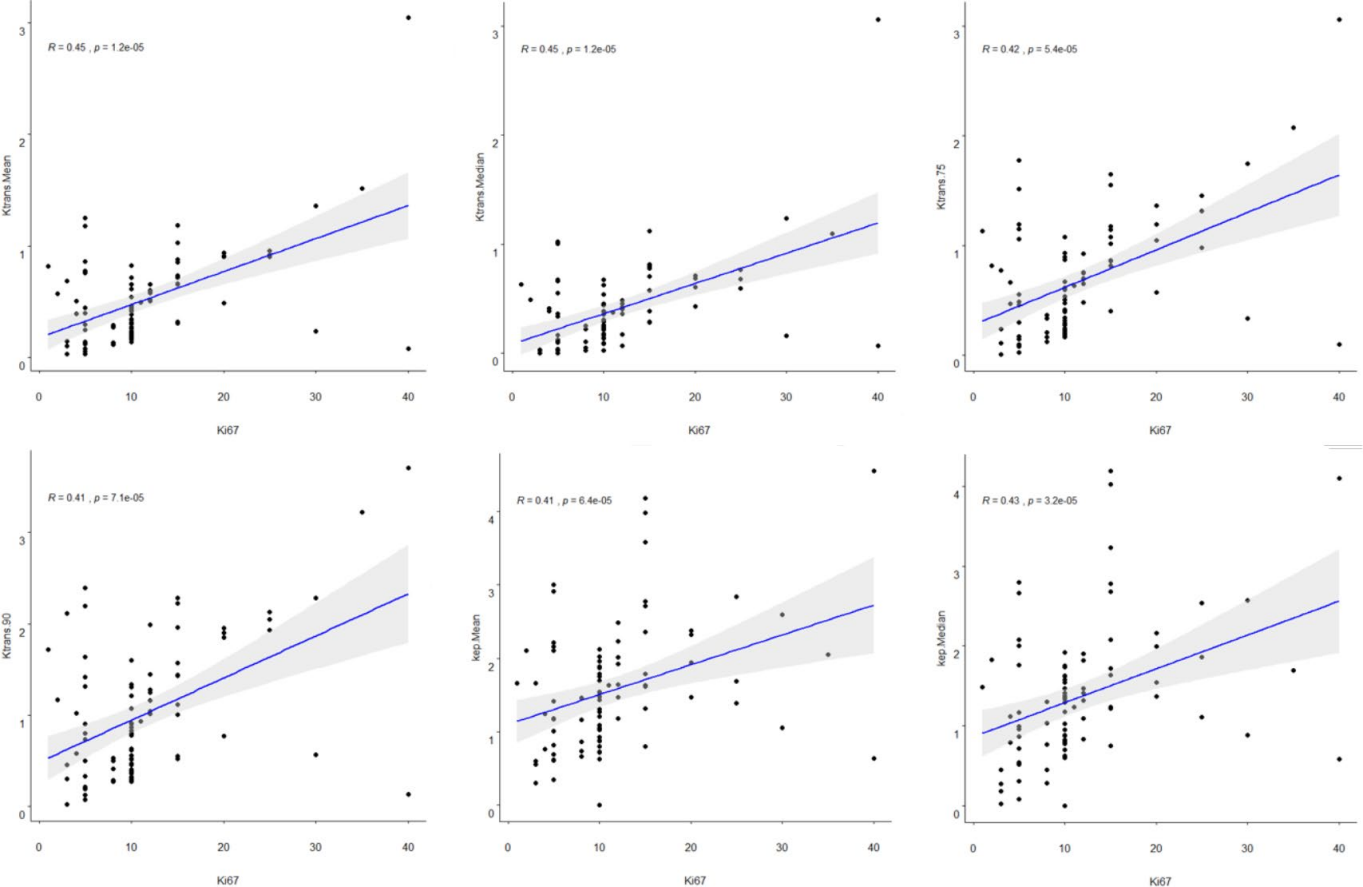
The correlation scatter plot between six selected histogram parameters including K^trans^‐based median, mean, 75th,and 90th percentile and K_ep_‐based median, mean and Ki‐67 expression level in prostate cancer patients, in which both median and mean of K^trans^ had the highest positive correlation (*r* = 0.450, *p *< 0.05)

The correlation of histogram features for DCE‐MRI pharmacokinetic parameters with Ki‐67 expression of PCa was demonstrated in Table 2. The median, mean, 75th, and 90th percentile derived from K^trans^ and K_ep_ had a moderately positive correlation with Ki‐67 expression (*r* = 0.361~0.450, *p *< 0.05), in which both median and mean of K^trans^ had the highest positive correlation (*r* = 0.450, *p *< 0.05) (Figure 3). The median, mean, 25th, 75th, and 90th percentile derived from V_e_ had a weakly positive correlation with Ki‐67 expression (*r* = 0.247~0.316, *p *< 0.05). All V_p_‐based histogram parameters except area had a weakly negative, or lack of correlation with Ki‐67 expression.

**TABLE 2:** Correlation of histogram features for DCE‐MRI pharmacokinetic parameters with Ki‐67 expression in prostate cancer patients

Histogram parameters	K^trans^		K_ep_		V_e_		V_p_	
	*r*	*p*	*r*	*p*	*r*	*p*	*r*	*p*
Minimum	0.080	0.460	0.141	0.190	0.079	0.463	−0.139	0.196
Maximum	0.392	0.000*	0.356	0.001*	0.160	0.137	−0.020	0.854
Median	0.450	0.000*	0.430	0.000*	0.316	0.003*	−0.277	0.009*
Mean	0.450	0.000*	0.410	0.000*	0.310	0.003*	−0.175	0.104
Area	0.230^^^	0.031*	0.222^^^	0.037*	0.197	0.066	0.222^^^	0.037*
10%	0.241^^^	0.024*	0.220^^^	0.040*	0.206	0.054	−0.323	0.002*
25%	0.283	0.008*	0.298	0.005*	0.247^^^	0.020*	−0.325	0.002*
75%	0.420	0.000*	0.399	0.000*	0.290	0.006*	−0.189	0.078*
90%	0.410	0.000*	0.361	0.000*	0.247*	0.020*	−0.102	0.345

Correlation is significant at the ^^^0.05 level, whereas the remaining correlation is significant at the 0.01 level.

* *p* value < 0.05.

**FIGURE 4:**
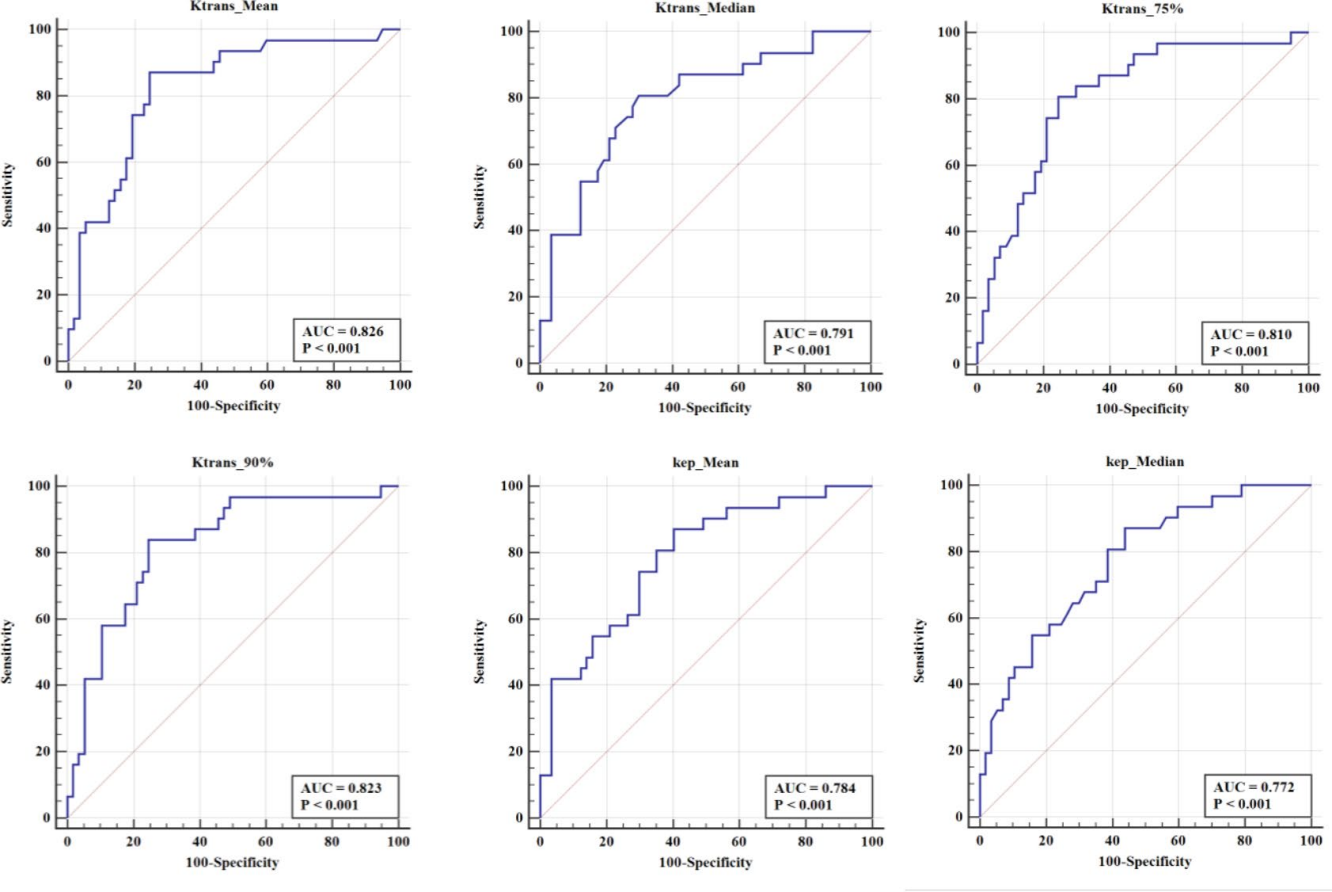
The receiver operating characteristic curve of six selected histogram parameters including K^trans^‐based median, mean, 75th, and 90th percentile and K_ep_‐based median, mean for discriminating the high Ki‐67 expression from the low Ki‐67 expression in prostate cancer. The area under the receiver operating curve (AUC) was presented. The AUC of the K^trans^‐based mean was the highest (0.826)

We chose the histogram quantitative parameters with an *r* value ≥0.4 to discriminate the high Ki‐67 expression from the low Ki‐67 expression PCa. And the diagnostic efficacy of six selected histogram parameters including the K^trans^‐based median, mean, 75th, and 90th percentile and K_ep_‐based median, mean was assessed by ROC curve and shown in both Table 3 and Figure 4. The AUC of the K^trans^‐based mean was the highest (0.826). When the cut‐off of K^trans^‐based mean was ≥0.47/min, its Youden index, sensitivity, and specificity were 0.625, 0.871, and 0.754, respectively. The AUC of the K_ep_‐based median was the lowest (0.772).

**TABLE 3:** The diagnostic efficacy of DCE‐MRI histogram parameters for discriminating the high Ki‐67 expression from the low Ki‐67 expression prostate cancer

Parameters	Cut‐off	AUC	Youden index	Sensitivity	Specificity
K^trans^ median	≥0.360	0.791	0.508	0.806	0.702
K^trans^ mean	≥0.470	0.826	0.625	0.871	0.754
K^trans^ 75%	≥0.630	0.810	0.56	0.806	0.754
K^trans^ 90%	≥0.920	0.823	0.593	0.839	0.754
K_ep_ median	≥1.070	0.772	0.432	0.871	0.561
K_ep_ mean	≥1.310	0.784	0.467	0.871	0.596

## 4 DISCUSSION

This study sought to investigate whether preoperative quantitative parameters of DCE‐MRI using histogram analysis can assess the Ki‐67 expression for PCa patients. We found that the median, mean, 75th percentile, and 90th percentile derived from K^trans^ and K_ep_ had a moderately positive correlation with Ki‐67 expression. We also chose the histogram quantitative parameters with an r value ≥0.4 to discriminate high Ki‐67 expression from low Ki‐67 expression in PCa. And six histogram parameters derived from K^trans^ and K_ep_ were selected, in which the AUC of mean for K^trans^ was the highest (0.826). Thus, the results shown herein indicate that the histogram analyses of DCE‐MRI quantitative parameters are correlated with Ki‐67 expression, which has the potential to noninvasively assess the expression of Ki‐67 in patients with PCa.

Several studies have previously evaluated the correlation between DCE‐MRI and histopathological parameters, predominantly focusing on microvessel density and Gleason score.^11^, ^12^, ^24^, ^25^ Vos et al. found that the K^trans^, K_ep_, and statistics of wash‐in had significant differences between patients with low, intermediate‐grade, and high‐grade PCa.^11^ Niekerk et al. correlated between pharmacokinetic parameters of DCE‐MRI and histopathologic microvascular and lymphatic parameters in organ‐confined PCa. This study suggested that K_ep_ had a significant correlation with microvessel density and perimeter by analyzing the ratio between tumor and normal tissue.^12^ However, this study only included 18 PCa patients with pT2a tumors. In another study assessing the correlation of DCE‐MRI with microvasculature density and Gleason scores using a computer‐extracted approach, Singanamalli et al. found that the extracted features including washout gradient and enhancement ratio were correlated with microvessel architecture and discriminated low from intermediate and high GS PCa.^24^

This study sought to compare pharmacokinetic parameters of DCE‐MRI with Ki‐67 using a novel histogram analysis and is therefore original to the previous studies. The correlation of the means and medians of K^trans^, K_ep_, and V_e_ against Ki‐67 expression indicated that the median was greater than the mean. Therefore, it was suggested that the uneven growth and complexity of microvasculature in PCa lesions raise with the increase in malignancy. Therefore, the mean and median must be considered together in determining the aggressiveness of a PCa lesion.

Using the Ki‐67 expression a proliferation marker of tumor cells is an independent predictor for unfavorable pathologic outcomes and biochemical recurrence.^3^, ^4^, ^5^ Among the four quantitative parameters of DCE‐MRI, K^trans^ and K_ep_ had a better correlation with Ki‐67 expression. The quantitative parameters of DCE‐MRI can provide information about different aspects of the tumor angiogenesis and permeability, for example, K^trans^ is a reliable parameter defined as the rate of the contrast agent transferring from the blood to the extravascular extracellular space (EES), reflecting a combination of vessel density and permeability.^12^, ^14^, ^15^, ^17^ Contrary to K^trans^, K_ep_ represents the rate of the contrast agent transferring from the EES back to the blood. PCa demonstrates tumor angiogenesis. The new tumor vessels are created with thin angiogenesis, highly permeable, and irregular shape, structure, and organization.^26^, ^27^ The elevated Ki‐67 level, an important component of histologic grade and stage, is commonly considered to greater tumor proliferative potential in PCa. Such tumors seem to have high cellularity and may, consequently, show higher K^trans^ and K_ep_.

There was a minimal correlation between the histogram parameters derived from V_e_, V_P_, and Ki‐67. Ve predominantly reflects the amount of contrast agent that is temporarily trapped in the EES.^12^, ^17^ This is likely explained that the effect of V_e_ can be weakened when the neovascularization density of the tumor site is higher than that of surrounding normal tissues. The PCa is usually full of high cell density regions. There are still some deficiencies in using V_e_ as a quantitative parameter to reflect the vascular condition of tumors.^28^ V_P_ predominantly reflects the percentage of contrast agents in the blood.

Recently, several studies have investigated the correlation between histogram parameters derived from DCE‐MRI and Ki‐67 in other solid tumors.^14^, ^17^, ^22^, ^29^ Surov et al. observed that histogram‐based parameters skewness, kurtosis, and entropy of K^trans^, K_ep_, and V_e_ can be used as markers for proliferation activity (Ki‐67) in head and neck squamous cell carcinoma.^14^ Additionally, Jain et al. found that DCE‐MRI was associated with Ki‐67 in glioma.^29^ Interestingly, Nagasaka et al. observed that the mean and the coefficient of V_e_ were correlated with Ki‐67, whereas histogram parameters of K^trans^ and K_ep_ were lack in association with Ki‐67 in breast cancer.^17^ Our results found that the maximum, median, mean, 75th, and 90th percentile of K^trans^ and K_ep_ had a better correlation with Ki‐67 expression than that of V_e_ in PCa. This discordance may be caused by intertumoral heterogeneities, differences in the study populations.

This study has several limitations. First, this is a retrospective and small sample study. Therefore, a prospective study with a large sample size is necessary to validate our primary findings. Second, the transitional and peripheral zones of PCa were not distinguished as some lesions were too large to be partitioned. Furthermore, patients with a lesion diameter of less than 5 mm on DCE‐MRI were excluded from this study because it was not possible to delineate tumor regions during postprocessing. This may cause patient selection bias. Despite the limitations mentioned above, the integrity and significance of the data still provided a strong case for the relationship between the DCE‐MRI histogram and the Ki‐67 expression level.

## 5 CONCLUSIONS

In conclusion,this work demonstrated a correlation between pharmacokinetic parameters of DCE‐MRI and Ki‐67 expression by performing a histogram analysis in PCa patients. The quantitative parameters, of K^trans^ and K_ep_, are strongly correlated with Ki‐67 expression, which has the potential to noninvasively assess the expression of Ki‐67 in patients with PCa. In the future work, a whole‐mount pathological specimen will be adopted as the reference to correlate DCE‐MRI quantitative parameters with Ki‐67 expression in PCa.

## CONFLICT OF INTEREST

PP was employed by GE Healthcare Life Sciences. The remaining authors declare that the research was conducted in the absence of any commercial or financial relationships that could be construed as a potential conflict of interest.

## ETHICAL APPROVAL

This retrospective study was approved by our institutional review board and informed consent requirement was waived.

## Supporting information

Table S1

## Data Availability

The data that support the findings of this study are available from the corresponding author upon reasonable request.
