# Supplementary material for: Preoperative histogram parameters of dynamic contrast‐enhanced MRI as a potential imaging biomarker for assessing the expression of Ki‐67 in prostate cancer
Source: Cancer Med. 2021 Jun 12;10(13):4240–9. doi: 10.1002/cam4.3912 (PMC8267123; doi:10.1002/cam4.3912)
Supplement: Supplementary file 1 — Table S1 [file CAM4-10-4240-s001.docx]

Supplementary Material

# 1 Supplementary Tables

**Supplementary Table 1**. These details of the imaging sequence parameters

| Parameters | T1WI | T2WI | DWI | DCE |
| --- | --- | --- | --- | --- |
| Sequence | FSE | FSE | EPI | LAVA |
| TR/TE (ms) | 795/11.2 | 12900/100 | 3541/67.3 | 3.7/1.4 |
| Slice thickness (mm) | 5 | 3.5 | 3.5 | 3.5 |
| Gap (mm) | 1 | 1 | 1.5 | 0 |
| FOV (cm) | 40×4 | 18×20 | 38×32 | 40×40 |
| Matrix | 320×192 | 288×288 | 128×128 | 200×160 |
